# Integrated Flow Chamber System for Live Cell Microscopy

**DOI:** 10.3389/fbioe.2019.00091

**Published:** 2019-05-01

**Authors:** Carlo Kriesi, Martin Steinert, Anastasios Marmaras, Claudia Danzer, Virginia Meskenaite, Vartan Kurtcuoglu

**Affiliations:** ^1^TrollLABS, Department of Mechanical and Industrial Engineering, Norwegian University of Science and Technology, Trondheim, Norway; ^2^The Interface Group, Institute of Physiology, University of Zurich, Zurich, Switzerland

**Keywords:** cell culture, mechanobiology, fluid shear stress, flow chamber, device, integrated system

## Abstract

*In vitro* quantification of the effect of mechanical loads on cells by live microscopy requires precise control of load and culture environment. Corresponding systems are often bulky, their setup and maintenance are time consuming, or the cell yield is low. Here, we show the design and initial testing of a new cell culture system that fits on standard light microscope stages. Based on the parallel plate principle, the system allows for live microscopy of cells exposed to flow-induced shear stress, features short setup time and requires little user interaction. An integrated feedback-controlled heater and a bubble trap enable long observation times. The key design feature is the possibility for quick exchange of the cultured cells. We present first test results that focus on verifying the robustness, biocompatibility, and ease of use of the device.

## Introduction

*In vivo*, cells are exposed to mechanical stress in various ways (Ho et al., 2018), and exhibit cell type specific reactions to those (Davies and Tripathi, 1993). One well characterized example is how blood flow induced shear stress acts on vascular endothelial cells, thereby modulating vascular permeability (Orsenigo et al., 2012), which results in e.g., arterial blood pressure control (Wang et al., 2016). Since it is not possible to study mechanical influences in an isolated manner *in vivo*, various devices have been developed to expose cells to corresponding stimuli *in vitro*. These devices follow different fundamental principles, for instance: Mechanical deflection by the means of e.g., flexible silicone wells (Matsui et al., 2018); Flow-induced shear stress in e.g., parallel plate flow chambers (Koslow et al., 1986; Usami et al., 1993; Levitan et al., 2000) or under rotating discs (Chakraborty et al., 2016); Vibration-induced flow (Xu et al., 2018); The combination of microfluidic array and mechanical stimuli (Ho et al., 2018).

In this article, the focus lies on devices that rely on flow-induced shear stress at the cell's surface (wall shear stress or WSS). Such devices have enabled insights into for instance: How WSS activates molecular pathways in mouse embryonic stem cells (Illi et al., 2005); The impact of WSS on cell migration during wound healing (Franco et al., 2013); Cancer cell viability under WSS (Fan et al., 2016).

One popular design relies on flow between two parallel plates (Frangos et al., 1985). This design allows for well-defined, uniform shear stress on the cultured cells (Nauman et al., 1999), and also allows, in principle, for concurrent live light microscopy. One important shortcoming of current parallel plate devices is their incompatibility with high-throughput setups. This is a reflection of the time consuming, complex startup preparations needed to avoid leakage and air bubbles in these generally “bulky systems” (Salek et al., 2012). Parallel plate devices contrast with the simple approach of placing multi-well plates on an orbital shaker to induce shear stress by swirling culture medium. The latter approach allows for high throughput, but exposes cells to inhomogeneous mechanical stresses dependent on various factors, including the amount of medium in the respective well (Salek et al., 2012). Furthermore, concurrent live microscopy is not possible. Another prominent approach relies on microfluidic devices that can be rapidly prototyped (Duffy et al., 1998) and allow for high throughput, since they can offer a high spatial density of flow paths (Thorsen et al., 2002). Despite the small form factor of microfluidic devices, the required pumps and heaters result nevertheless in bulky systems. In addition, boundary effects create inhomogeneous WSS across microchannels. Microfluidic devices further reach their limit when larger structures such as organoids need to be investigated or when mechanical access to the cells is required, e.g., in wound healing assays (Liang et al., 2007).

With the existing solutions in mind, the authors developed an integrated flow chamber system, subsequently referred to as “the device,” that adopts strengths of current approaches while removing shortcomings, thereby allowing rapid, well-controlled studies of cells exposed to mechanical stimuli using live microscopy. This vision implied certain design constraints: First and foremost, the “bulkiness” of a classic parallel plate flow chamber setup, as Salek et al. (2012) put it, or any setup for that matter, had to be reduced without sacrificing accuracy and control of the imposed mechanical stimulus. One crucial element of *bulkiness* is related to the central challenge that all systems have in common, namely maintaining a temperature hospitable to the cells. Either a temperature-controlled containment for the entire microscope and its immediate surroundings must be provided, or the cell culture medium must be accurately preheated before it reaches the cells. The first approach allows for installing any equipment within the containment as long as it fits space wise. However, newly introduced elements take time to reach the target temperature, making this approach inefficient in shared microscope settings. In such an environment, the second approach allows for higher throughput, provided that compatibility with existing equipment, in particular with microscopes, is maintained. To further decrease down time, cells should be replaceable quickly, e.g., to change to a control group while maintaining experimental conditions, or to switch to a different type of experimental setup without extensive reconfiguration. Herein, we describe the development and testing of such an integrated flow chamber system.

## Materials and Methods

### Design Goals and Implementation

The overarching aim throughout the development was a device usable by researchers trained in cell culture work, but without prior experience in flow cultures. This implies *soft* requirements of strong focus on user-centered design and subsequent ease of use. The technical constraints mentioned in the Introduction were translated to the following four main *hard* design requirements:

- The device must include a heating system that maintains the culture medium at a specific temperature and reacts rapidly to perturbations.
- The flow chamber and the cultured cells must be exchangeable without the use of tools.
- The complete system must be compatible with and fit on a standard light microscope stage.
- The device must be biocompatible in the sense that it does not harm the growth of cells and can be cleaned to remove contaminants.

**Figure 1:**
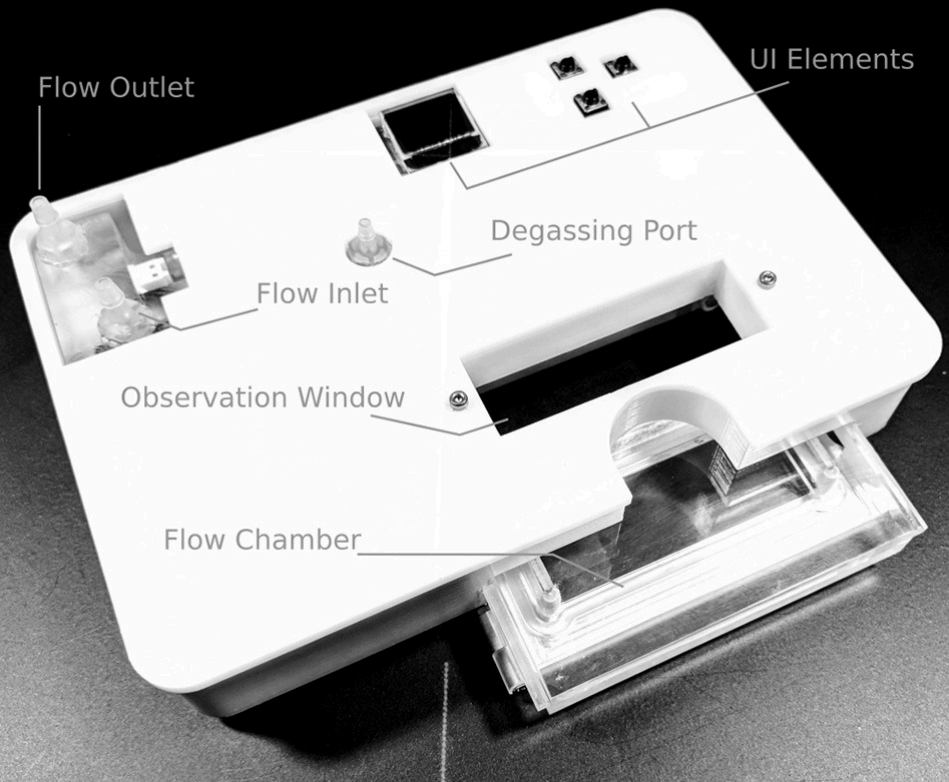
Overview of the device. The in- and outlet ports connect to the heater and to the cell culture medium container, respectively. The degassing port is part of the bubble trap, located ahead of the flow chamber where the cells are located. The user interface (UI) elements, namely a 64 × 48 pixel OLED display and three buttons, allow the user to set the desired flow rate and temperature. During operation, the current outlet temperature, heater status, and possible sensor errors are displayed.

The development work included multiple prototype-test-iterate rounds according to the Wayfaring principle (Steinert and Leifer, 2012), where each iteration was performed to answer specific design questions and subsequently used for guiding the focus of further development (Leifer and Steinert, 2011). The final design is shown in Figure 1.

#### Device Overview

Addressing the goal of physical compatibility with standard inverted microscope stages, the device has a footprint of 160 × 110 mm^2^, and overall height of 36 mm, which is low enough for positioning under the condenser of the microscope. Within this building space, the device includes:

- User interface (UI), consisting of an OLED display and three buttons.
- Heater including two temperature sensors.
- Degassing chamber.
- Flow chamber with optical access.
- Electronic circuitry and microcontroller for regulating temperature, as well as handling the in-/output stream for the UI.

**Figure 2:**
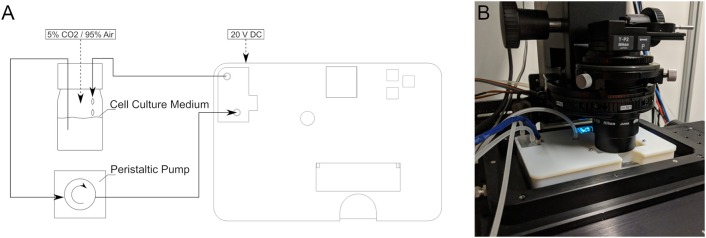
**(A)** Schematic of the complete experimental circuit with the device. **(B)** The device in use on an inverted microscope.

The setup in a general use case is as follows (Figure 2): The cell culture medium flows driven by an external peristaltic pump from an external reservoir into the device. Therein, it flows past the inlet temperature sensor, through the heater, past the outlet temperature sensor and via the bubble trap into the flow chamber and back to the reservoir. The total liquid volume within the device is 20 ml.

#### Production Methods

Apart from some standard components such as connectors and resistors, everything was built in-house at the prototyping facility TrollLABS at the Norwegian University for Science and Technology (NTNU, Trondheim, NO). Namely, the production relies on a laser cutter (Gravograph LS1000 XP, Gravotech Marking SAS, Rillieux-la-Pape, FR), a high-end 3D-printer (Objet30, Stratasys, Rehovot, IS, in combination with the same company's VeroWhitePlus material), a CNC mill (Mazak VCN-705E, Mazak corp., Florence, KY, US), as well as soldering equipment.

#### Heater and Power Control

**Figure 3:**
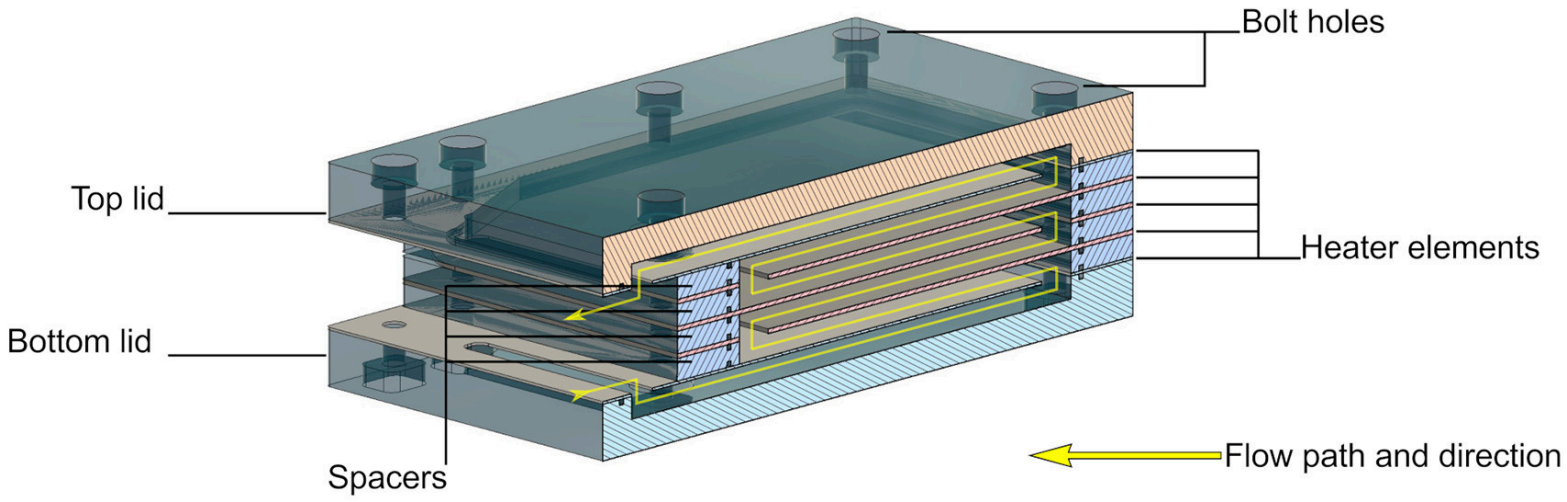
Cross-section of the CAD model of the heater assembly showing how the five heating elements are arranged to maximize heated area per volume.

**Figure 4:**
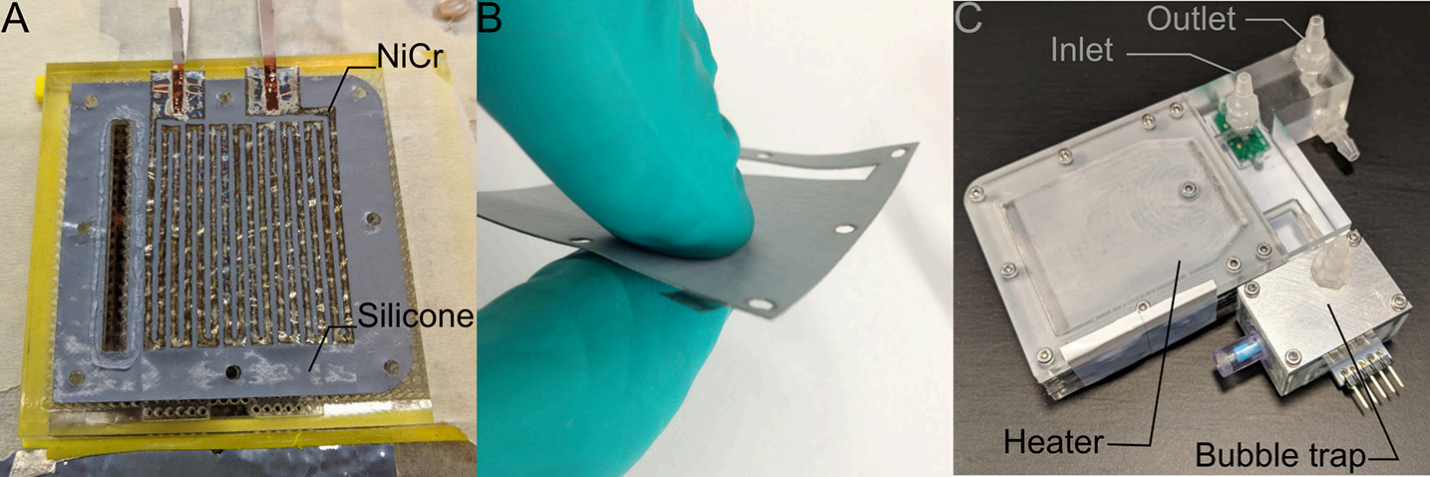
**(A)** The heating elements are based on laser-cut NiCr foil sandwiched between two silicone foils. **(B)** The result is a watertight heating element with 0.45 mm thickness. **(C)** The complete heater assembly includes the in- and outlet ports, five heating elements, as well as the bubble trap upstream of a valve that leads to the flow chamber. The green plate visible under the inlet port is one of the two temperature sensor breakout boards (the second one, not visible in this picture, is located within the bubble trap).

The general design principle of the heater is that of a parallel plate heat exchanger, where each plate is a heating element made of a laser-cut nickel-chrome (NiCr) foil of 0.01 mm thickness, sandwiched between two laser-cut KU-EGF20 silicone foils (Aavid, Laconia, NH, US) of 0.22 mm thickness. The overall thickness of one heating element is thus 0.45 mm. This design creates a large surface area needed for homogenous temperature distribution within the cell culture medium within a range of flow rates. It also mitigates the risk of local overheating, which would result in medium denaturation. All other structural elements, namely the top and bottom lids as well as the spacers, are made of acrylic. The lids are CNC milled, whereas the spacers are laser cut. A CAD model cross-section of the heater assembly with annotations is presented in Figure 3. The entire structure is held together by ten M2 bolts, allowing for an even clamping force necessary to avoid leakage. The heating power depends on the applied voltage, as well as on the resistance of each element that can be adjusted by changing the pattern of the NiCr foil. In the current design, a single heater element has an average resistance of 47 Ohm. Therefore, following Ohm's law, the five heaters in parallel have a total resistance of 9.6 Ohm, yielding a maximum power output of roughly 40 Watts at 19.5 V. Theoretically, this is sufficient to increase the temperature of water flowing at up to 35 ml/min by 16 Kelvin, assuming adiabatic conditions and constant heat capacity of 4.2 kJ/kgK. Attached to the heater assembly is the distribution block that not only guides the flow of the cell culture medium in and out of the heater, but also includes the bubble trap. The laser-cut NiCr foil, as well as the complete heater including the distribution block, are depicted in Figure 4.

Sensirion STS30 temperature sensors (Sensirion AG, Staefa, CH) with a resolution of ± 0.2°C are located at the inlet and outlet of the heater. The inlet sensor enables rapid adjustment to thermal changes in the inlet and surroundings and allows for a substantially accelerated warm-up procedure by feed-forward control that relies on known flow rate, inlet temperature, and heater power. The outlet sensor is used for fine-tuning the outlet temperature to the target value by feedback control. The sensor data are processed by the control system running on a microcontroller that adjusts the heater power through a MOSFET transistor. This transistor is controlled by pulse width modulation with 8-bit resolution (256 steps), resulting in a power adjustment resolution of 0.16 W/step. By fluctuating the power level, the heater can precisely adjust to the target temperature.

#### Degassing

Gas bubbles within the flow chamber must be avoided, as they can damage cells and alter the mechanical stimulus. Since the solubility of the most prevalent gases in the air generally decreases with increasing temperature (Battino and Clever, 1966), gas bubbles are likely to form in the heater. Therefore, the degassing unit is placed immediately downstream of the heater and immediately upstream of the flow chamber (see Figure 4). The degassing unit consists of two chambers that are separated by a polytetrafluoroethylene (PTFE) membrane (TRACE Analytics, Braunschweig, DE). Its top side is in direct contact with surrounding air at atmospheric pressure and its bottom side is in contact with the medium. Gas bubbles that may develop rise within the medium up to the PTFE membrane. These gas bubbles are at the same pressure level as the surrounding medium, and thus at a slightly higher pressure than atmosphere. The actual gas removal is, therefore, a diffusion process driven by the gas partial pressure difference across the membrane (Cussler, 2009). Furthermore, the bubble trap works completely passively and is self-regulating: An increase of the flow rate implies both a larger volume of gas that is transported into the system and needs to be removed, as well as a higher gas pressure drop across the membrane that increases diffusion. This is because the cell culture medium pressure in the degassing unit increases with increasing flow rate.

#### Electronics

The electronic system is based on an open source Arduino Micro microcontroller (Arduino.cc). It is integrated in the electronic circuitry of the device consisting of three connected printed circuit boards (PCBs):

- The *main PCB* includes the microcontroller, MOSFET, and necessary connectors for power distribution.
- The *sensor PCB* connects both temperature sensor breakout boards and carries the necessary resistors and capacitors.
- The *display PCB* contains the UI elements and their resistors.

The microcontroller handles temperature control as well as input from and output to the UI. It also ensures shutdown of the heater in case of faulty temperature sensors.

#### Flow Chamber

**Figure 5:**
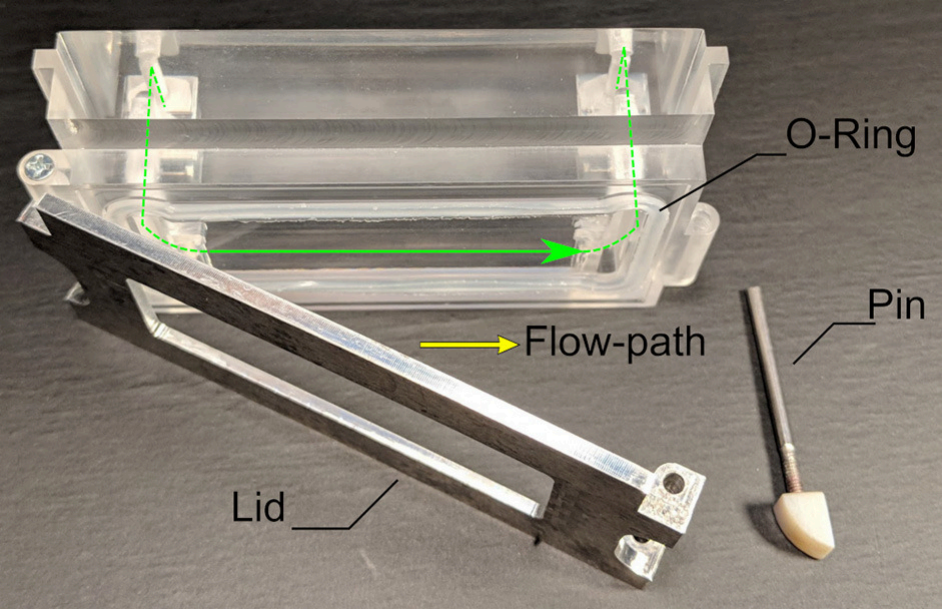
The flow chamber that was used in all tests. The lid can hold a 38 × 76 mm^2^ glass slide of 1 mm thickness on which the cells are attached. Closing the lid with the pin creates a rectangular flow-channel of 0.3 × 20 mm^2^ cross-section, sealed off by the silicone O-ring.

Overall, the flow chamber is a cassette-like insert as shown in Figure 1. It consists of a milled acrylic body and an aluminum lid that can hold a 38 × 76 mm^2^ glass slide of 1 mm thickness on which cells are cultured in a regular incubator before it is placed in the flow chamber. A specifically designed, cast silicone O-ring made of Ecoflex 00-50 (Smooth-On, Macungie, PA, USA) seals the flow channel. The locking mechanism is a single pin that can be removed and inserted by hand. Figure 5 provides a detailed view of the chamber and its individual parts. For the tests reported herein, a rectangular channel of 0.3 × 20 mm^2^ cross-section was used. With the insertable glass-slide forming the bottom and the acrylic cassette the top, this corresponds to a parallel flow chamber setup. The level of shear stress τ [Pa] acting on the cells can be approximated as

(1) $$\begin{array}{r} \tau =\frac{6Q\mu }{wh^{2}}, \end{array}$$

where *Q* is the flow-rate [m^3^/s], μ is the dynamic viscosity [Pa s], and *w* [m] and *h* [m] are channel width and height, respectively (Levitan et al., 2000). The tests reported herein were run with flow rates between 5 ml/min and 20 ml/min, resulting in shear stresses between 0.25 Pa and 1 Pa. These numbers were further validated by computational fluid dynamics (CFD) analyses (see section Flow and WSS Analysis).

### Validation of Design Goals

The design goals are related to user interactions (*soft* goals) and to robustness for live cell experiments (*hard* goals). Attainment of the soft goals was validated by observing the interactions of researchers with the device, and by analyzing their feedback on its usability. Attainment of the hard goals was validated by tests performed in various settings and surroundings, with and without cells, to check for robustness, heater performance, and biocompatibility.

#### Ease of Use

As mentioned in the introduction, “bulkiness” had to be reduced. A proxy for the reduction in bulkiness and, therefore, for the increase in ease of use, is the number of tools and components required to run the *in vitro* flow experiments. This, in turn, is associated with the time needed to set up an experiment, which we use here as a surrogate for ease of use. Since individual research groups follow different protocols and do not use the same pump or other auxiliary equipment, our reported values only include the time the user spends in direct interaction with the device.

#### Robustness

Operation with expensive inverted microscopes requires robustness with respect to device integrity. In particular, leak prevention and fail-safe mechanisms must be robust, while maintaining compatibility with the microscope. Experiments with long run times require robust heating and temperature control, and day-to-day use necessitates hassle-free cleaning, loading and unloading, even as the device ages. To validate robustness, we have successfully tested the device for several 100 h under a variety of operating conditions.

#### Test Preparations

Device surfaces exposed to the cell culture medium are made of very stable plastics (HDPE and Acrylic) and silicones. The medium does not come in contact with any metals. All of the used materials can handle regular disinfection liquids such as 1–3% H_2_O_2_ and 70% ethanol. To ensure biocompatibility, we performed tests with several cell types, including sensitive primary cells as listed in Table 1. The following cleaning protocol was followed in preparation of the tests:

1) Flush with 70% ethanol for 15 min.
2) Drain system.
3) Flush with double distilled H_2_O.
4) Drain system.
5) Flush with 3% H_2_O_2_ for 20 min.
6) Drain system.
7) Dry system by forced airflow.

**Table 1:** Overview of cell types and shear stress levels used in the validation tests.

**Cell Type**	**Origin**	**WSS [Pa]**
Primary glia	Prepared from C57B6/JjRj mouse, Janvier Labs (Le Genest-Saint-Isle, FR)	0.25
Primary neurons	Prepared from C57B6/JjRj mouse, Janvier Labs	0.25
Madin-Darby canine kidney cells (MDCK)	MDCK-GAP43-YFP	0.25
Human umbilical vein endothelial cells (HUVEC)	Thermo Fisher	1.00
Meningothelial cells (MEC)	BEN-MEN-1, DSMZ no.: ACC 599 (Leibniz-Institut DSMZ-Deutsche Sammlung von Mikroorganismen und Zellkulturen GmbH, Braunschweig, DE)	0.30

After cleaning, the whole system was pre-filled with cell culture medium containing antibiotics, namely 100 U penicillin and 0.1 mg streptomycin/mL (Sigma-Aldrich, St. Louis, MO, USA). The cells were cultured and passaged in the appropriate culture medium. For HUVEC, this was Medium 200 plus LSGS (Thermo Fisher, Waltham, MA, USA). For MEC, it was high glucose DMEM with GlutaMAX™ and sodium pyruvate (Thermo Fisher) supplemented with 10% FCS. For primary glial cells, DMEM plus GlutaMAX (Gibco, Thermo Fisher) was used. For primary neurons, Gibco neurobasal medium (Thermo Fisher) was used. Finally, for MDCK, DMEM D5796 (Sigma-Aldrich) was utilized.

For all tests, the cells were grown in an area created by a silicone ring on 38 × 76 mm^2^ glass slides until they reached the desired level of confluence (ideally 70–100%). Prior to placing the slide into the open lid of the flow chamber, the silicone ring was removed, and the glass slide was covered with additional medium to ensure that the cells were not exposed to air.

#### Test Procedure

Once the lid is closed and secured, the flow chamber is inserted into the prefilled main device. Two mechanically activated valves ensure that there is no leakage when the flow chamber is removed. After the flow chamber is inserted, the operator can power on the device, set a target temperature (here 37°C, maintained solely by the internal heater), and set the pump to the desired flow rate without turning it on. Finally, the device is placed on the inverted microscope stage, here on a Visitron Spinning Disk CSU-W1 (Visitron Systems GmbH, Puchheim, DE). The observation can start once the pump is turned on. The pH of the system was kept constant by gassing with 5% CO_2_/95% air into the reservoir.

In addition to testing biocompatibility with a broad variety of cells, the device was used for an explorative experiment, highlighting the capability of creating a hospitable environment, as well as enabling constant observation. It investigated the effect of WSS on MECs when grown on differently coated glass slides and exposed to WSS of 0.30 Pa.

## Results

### Heating and Temperature Control

**Figure 6:**
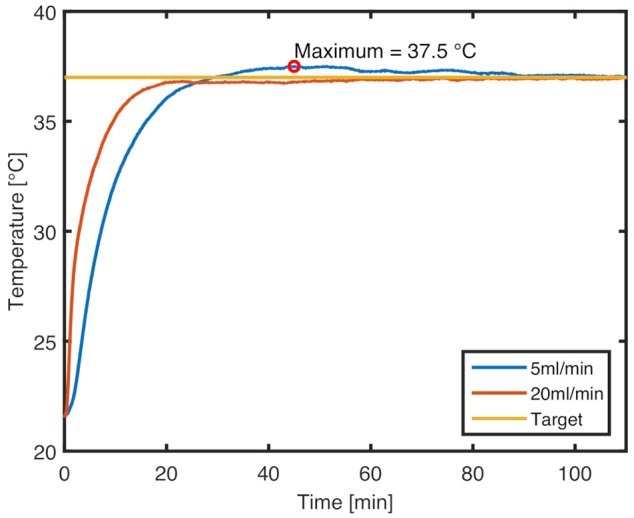
Characteristic temperature profiles for ramp-up to 37°C set temperature at flow rates of 20 and 5 ml/min, respectively. An overshoot of 0.5°C is seen at the lower flow rate.

The heating system proved capable of bringing the device to the desired output temperature rapidly and keeping it steady throughout the tests. Characteristic temperature profiles for ramp-up at 5 and 20 ml/min flow rate from 22°C to a target of 37°C are shown in Figure 6 (raw sensor data available in Supplementary Materials). The maximum overshoot at 5 ml/min, which corresponds to the lower limit of possible flow rates, is 0.5°C. At this flow rate, the target temperature is first reached at 29 min, and equilibrium is established at 90 min. At the upper flow rate limit of 20 ml/min, there is no overshoot, and the target temperature is reached within 21 min. In both cases, the temperature stabilizes on the target value, even if the cell culture medium reservoir heats up over time.

### Interactions

**Figure 7:**
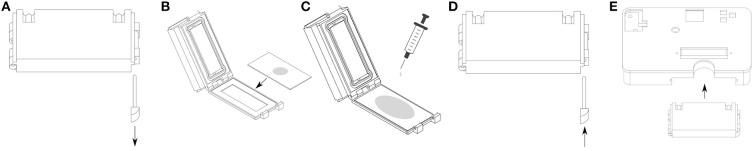
An overview of the user interactions when unloading and loading cells for an experiment. **(A)** Removing the pin opens the flow chamber. **(B)** The glass slide hosting the incubated cells is placed in the lid. **(C)** Extra cell culture medium is added. **(D)** Inserting the pin closes the flow chamber. **(E)** The flow chamber itself is inserted into the device.

No tools are required to operate the device. The individual steps of user interaction are as follows (see also Figure 2 for steps 1 and 2, and Figure 7 for steps 3–5):

1) A tube that connects to the reservoir via the peristaltic pump is attached to the inlet port of the device.
2) The outlet port is connected to a tube that drains either back to the reservoir or into a waste container.
3) The flow chamber is removed from the device and opened by removing the pin.
4) The glass slide with the cells is placed in the flow chamber and extra cell culture medium is added.
5) The flow chamber is closed by reinserting the pin and placed back in the device.
6) The power cord is connected to the device and the target temperature, as well as the utilized flow rate are set on the UI unit.
7) Once the experiment is done, the power cord is removed. The device can now be flushed, or the next culture slide can be inserted following the steps above.

The design ensures that neither the glass slide, nor the flow chamber, nor the pin can slide out while the device is in use, since they are physically blocked by the microscope stage. Multiple biologists have used the device accordingly, and a completely new user that had not interacted with the device before required minimal training (completed within 1 h) before being able to operate the device.

### Biocompatibility

**Figure 8:**
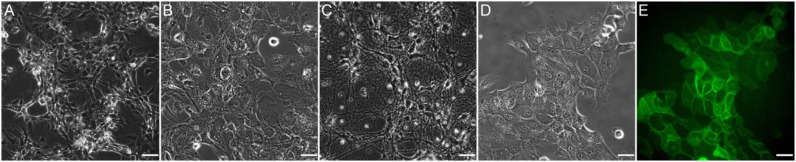
Images of the different cell types that were used for testing as listed in Table 1. **(A)** HUVEC; **(B)** Primary glia; **(C)** Primary neurons; **(D)** MDCK; **(E)** MDCK GFP signal. All images were taken <1 min from onset of flow and at 40x magnification. Scale bar: 35 μm.

The five cell types listed in Table 1 were exposed to shear stress they typically encounter *in vivo*. The cells underwent mitosis throughout the tests and behaved in line with what has been reported in the literature and/or what we have observed in previous experiments with standard parallel plate flow devices. Figure 8 shows five images that were recorded in these tests. Some cells survived for 72 h under flow (MEC), at which time the test was stopped, while others detached from the glass slide 30 min after onset of flow (glia). Overall, however, there were no signs of early cell death due to toxins or incompatible materials. Finally, the cleaning protocol listed in subsection Test Preparations was confirmed to be successful by observing the elimination of bacterial contamination within the device.

**Figure 9:**
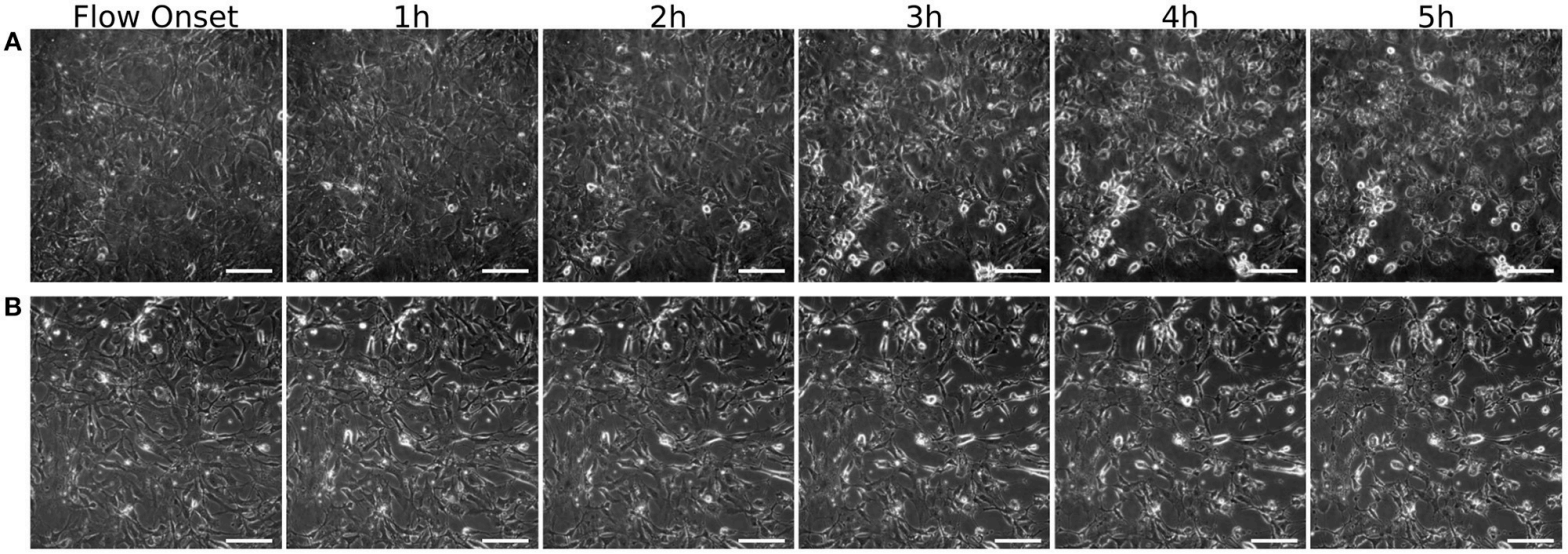
MEC grown on different coatings. Images are taken over 5 h at one image per minute at 40x magnification. Shown here are t=0 at the onset of 6 ml/min flow (0.3 Pa WSS), and cells after 1, 2, 3, 4, and 5h of shear exposure. **(A)** Poly-d-lysine coated glass slides and **(B)** fibronectin coated glass slides. Scale bar: 50 μm.

The results of an explorative test, comparing two coatings (Figure 9), show that the MEC cells grown on poly-d-lysine start rounding up and loosing attachment soon after the onset of flow. Grown on fibronectin, however, they mostly keep their initial morphology, at least up until the first 5 h of shear exposure. All images were recorded on the device described in this article.

### Flow and WSS Analysis

**Figure 10:**
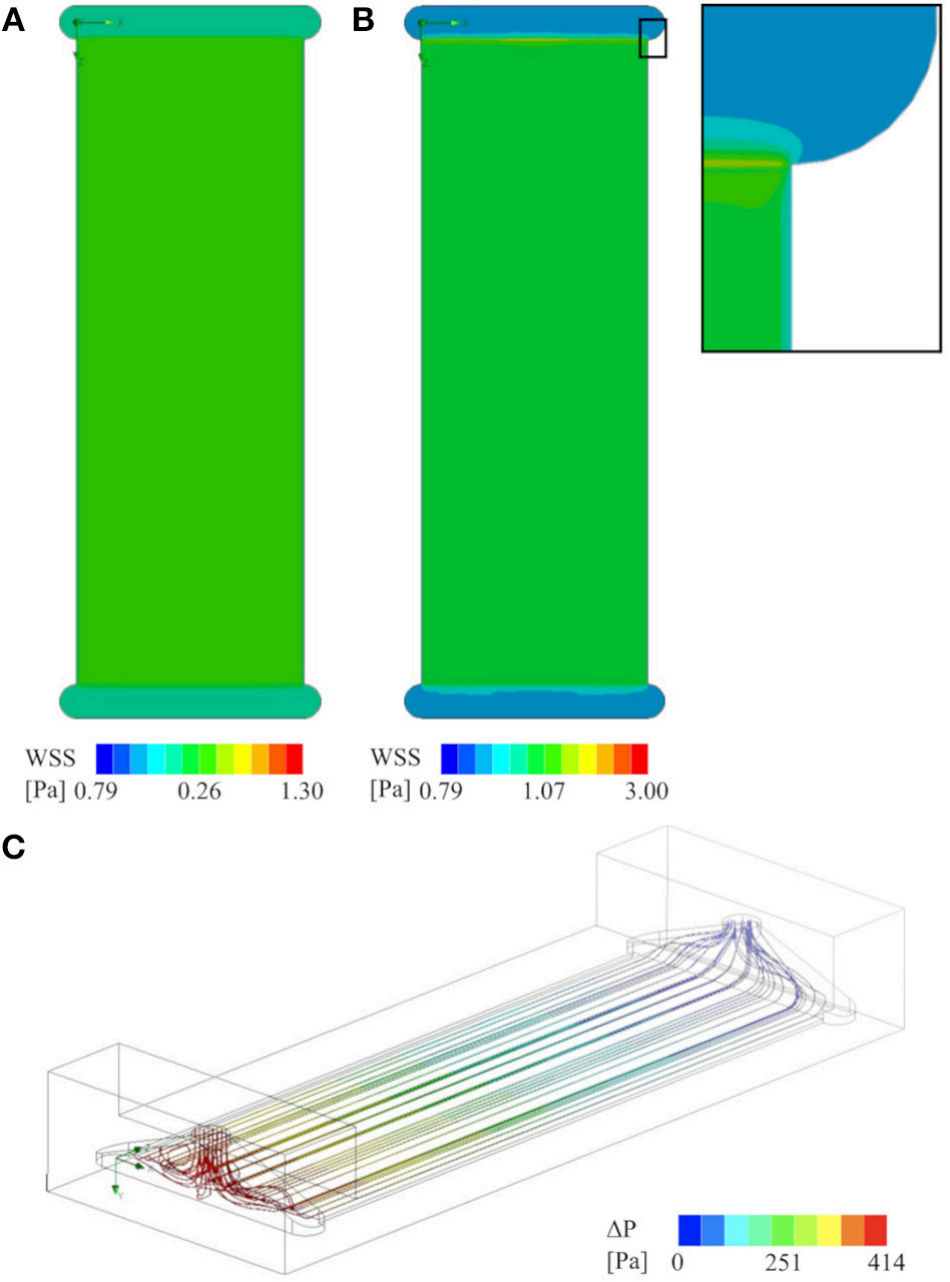
CFD analysis of the flow chamber that was used for all experiments (cross-section of 0.3x20 mm^2^): **(A)** Contour plot of WSS on the glass slide at 5 ml/min flow rate, and **(B)** at 20 ml/min, including a close up showing the boundary effects of the channel walls. **(C)** Stream lines visualizing the flow field from the inlet (red–high-pressure side) to the outlet (blue–low-pressure side) at 20 ml/min flow rate. There are no apparent zones of flow stagnation in the active area of the flow chamber.

In order to verify WSS values and shear stress homogeneity, as well as flow distribution, CFD analyses were conducted using SolidWorks Flow Simulation (Dassault Systèmes, Vélizy-Villacoublay, FR). They showed WSS of 0.26 Pa and 1.07 Pa on the surface of the glass slide at steady inlet flowrates of 5 ml/min and 20 ml/min, respectively. This, as well as streamlines through the flow chamber, are illustrated in Figure 10.

**Figure 11:**
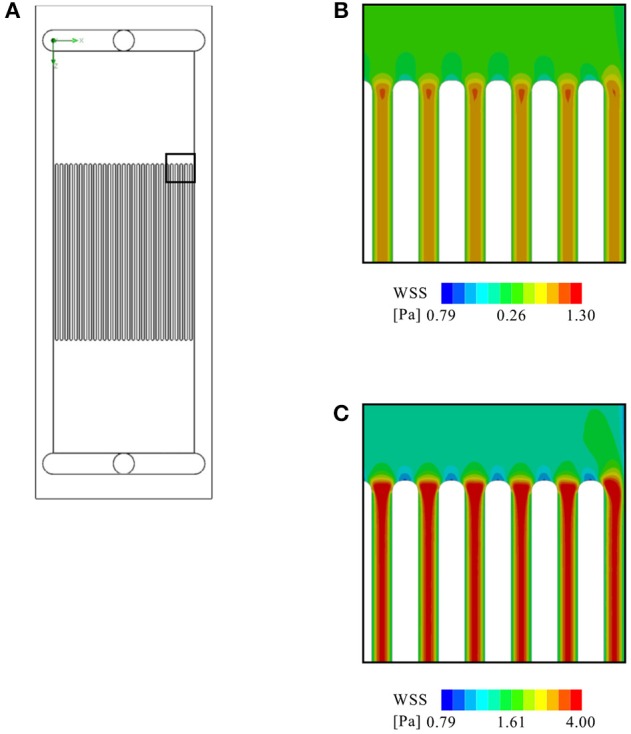
CFD analysis of a virtual microfluidic insert consisting of 30 evenly spaced channels (each with a cross-sectional area of 0.3 × 0.3 mm^2^): **(A)** The arrangement of the virtual microfluidic channels. Further shown are a close up of the contour plot of WSS on the glass slide at **(B)** 5 ml/min flow rate, and **(C)** at 20 ml/min.

To assess the compatibility of the device with microfluidics experiments, a microchannel insert was modeled and exposed to a simulated total flow rate of 5 and 20 ml/min, respectively. The virtual insert consisted of 30 evenly spaced channels, each with a cross-sectional area of 0.3 × 0.3 mm^2^. The results of this simulation are illustrated in Figure 11. The simulation shows that such an insert would yield a homogeneous flow distribution across all channels, yet inhomogeneous WSS distribution across individual channels due to boundary effects of the channel walls. Concretely, WSS ranges from 0.29–0.95 Pa and 1.15–3.8 Pa across each channel at the lower and higher flow rate, respectively, with the minimum values occurring close to the walls and the maximum in the channel center. The WSS determined in all simulations, as well as the corresponding pressure drops between inlet and outlet are listed in Table 2.

**Table 2:** Calculated WSS and pressure drop across the flow chamber.

**Channel cross-section [mm^**2**^]**	**Total flow rate [ml/min]**	**WSS on cells [Pa]**	**Pressure drop (inlet-outlet) [Pa]**
0.3 × 20 (1 channel)	5	0.26	103
0.3 × 20 (1 channel)	20	1.07	414
0.3 × 0.3 (30 channels)	5	0.29–0.95	295
0.3 × 0.3 (30 channels)	20	1.15–3.8	1189

## Discussion

This article introduces a device for flow-induced mechanical stimulation of *in vitro* cell cultures that includes a heater with controller, a bubble trap, and an insertable flow chamber. We have shown the results of first tests that focused on validating the robustness, biocompatibility, and ease of use of the device. Here, we will discuss to what extent the original design goals have been reached, and how the system can be further improved.

### Validation of Main Design Goals

The *hard* goals relevant for the fundamental functionality of the device have been met. The heating system allowed fast ramp-up with little temperature overshoot and maintained target temperature for the entire test duration. Denaturation of the cell culture medium due to overheating is, therefore, not a concern. In particular, the heating system allows exchanging medium containers or topping off medium without risking a temperature shock to the cells due to the feed-forward control loop. The accuracy of the temperature control is limited by the resolution of the temperature sensors (± 0.2°C), as well as by the 8-bit resolution of the controller. The resulting power resolution of 0.16 W/step at 20 V supply voltage translates to approximately 0.46°C/step at 5 ml/min flow rate. Given the lack of insulation around the heater, the heat loss to the surroundings have a significant effect, therefore enabling the temperature to stabilize with this setup. For lower flow rates, however, either the controller must be replaced with a more precise one to ensure more accurate temperature control, or the total heating power must be adjusted to lower levels.

CFD analyses confirmed the expected homogenous WSS distribution on the glass slide, and revealed no flow stagnation zones in the active area of the flow chamber. These results are in agreement with the findings of Nauman et al. (1999). Furthermore, the WSS levels calculated by CFD are close to the values obtained by approximation with Equation (1), with a maximum deviation of 7 %.

The virtual microfluidic insert creates a roughly three times higher pressure drop than the parallel plate flow chamber at the same flow rate. At the lower flow rate of 5 ml/min, this is not expected to create any issues. However, at the higher flow rate (20 ml/min), the pressure drop across the microfluidic array may be too high for the device to handle. One can, therefore, conclude that a microfluidic setup is feasible, but with certain limitations regarding the maximum flow rate, especially since with 0.3 × 0.3 mm^2^ the virtual microchannels still have a relatively large cross-section. A reduction in cross-sectional area would further increase the pressure drop. While the heater itself has enough power for a flow rate of up to 35 ml/min, the pressure drop at this flow rate across the flow chamber might cause leakage and/or ruptures along the flow path. Destructive testing will be required to determine the maximum pressure the system can withstand. Independent of the pressure-related limitations, microchannels never offers the same homogeneity of WSS across the cells as a larger parallel plate setup. This is due to boundary effects within individual channels, as visible in Figure 11.

The tests with live cells have proven that the device is compatible with the dimensional requirements of standard inverted microscope stages. It allows for continuous observation at 40x magnification, limited by the thickness of the glass slide that needs to withstand the internal hydraulic pressure without buckling. All observed behavior of the cells further indicates a healthy environment. In addition, the successful removal of a bacterial infection indicates that the cleaning protocol fulfills its task and, therefore, makes the device safely re-usable. While the explorative test comparing slide coating with poly-d-lysine and fibronectin was not intended to provide quantitative data on their relative performance, it does serve as a qualitative demonstration of how differences between two test settings can be optically observed. Given the fact that the device is compatible with phase contrast and fluorescent imaging techniques, analysis of metrics such as cell alignment over time as shown in e.g., Gong et al. (2017) is possible.

The *soft* design goals relating to user interaction and overall ease of use have also been reached. Integrating the heater, control system and degassing unit has reduced time required for setup compared to classic parallel plate devices. The *hard* design requirement of no tools plays into the ease of use of the whole device. If needed, the cells in the flow chamber can be exchanged within 2 min or the complete flow chamber can be exchanged in under 30 s. This is negligible compared to the duration of most flow experiments. In addition, the device also creates a safe environment for transporting cells to and from, e.g., cell culture and microscopy rooms. The small opening of the inlet and outlet parts are easily temporarily sealed. It is also possible to prepare multiple flow chambers for sequential microscopic imaging under flow, thus enabling higher throughput than with classic parallel plate systems.

### Vision

On a more abstract level, the device presents a platform that takes care of the well-being of cells, and the cassette-like flow chamber creates an experimental environment that can be adjusted to individual needs. Although the here presented flow chamber follows a parallel plate design, other chamber designs could be used as well. For instance, 3D-bioprinted structures could be perfused in the available space, and microfluidic structures could be integrated. Since the production of the structures itself, e.g., 3D-bioprinting, requires multiple hours if not days to begin with, a stable environment rather than high throughput is the main objective.

The device could become even easier to use if it required no external machinery and auxiliary devices at all. As shown in Figure 2, the device currently relies on an external pump and reservoir, as well as a gassing system. The enclosure offers some unused space that could be utilized to integrate a pump. However, this would also require integrated flow control, meaning that a flow-sensor and corresponding electronics would need to be added as well.

To compete with high-throughput setups, e.g., with ones based on orbital shakers, parallel operation would be required. This can be achieved to some extent by designing flow chamber inserts with multiple micro-channels, such as the virtual one analyzed herein. Together with the option of an injection port, the device could allow for drug testing on organoids connected in series, thereby taking in account possible *in vivo* intercommunications of the tissues upon drug intake.

The next level will be a system with automated cell culturing directly on the glass slides, as well as automated exchange of flow chambers, allowing to standardize and multiplex the research on the effect of fluid flow on cells and organoids. It seems unreasonable to assume that this approach will ever compete with the cost-efficient throughput of stacks of multi-well plates. However, the better control of mechanical stimuli and thus higher reliability of results may justify the higher cost of an automated parallel plate setup. Such a setup could be further used to connect multiple flow chambers, either in parallel to a central cell culture medium supply, or in series in order to observe downstream effects.

In conclusion, the device presented here is a flexible platform for the study of cells under flow-induced mechanical stimuli, potentially also with microfluidic inserts. Its key advantages are that it is easy to operate, time effective in its use, and biocompatible as indicated by tests with several cell types, including sensitive primary neurons.

## Author Contributions

All authors contributed ideas and concepts throughout the development of the device. CK did the physical implementation. VM, CD, AM, and CK performed the tests. MS and VK provided laboratories. All authors contributed and agreed to the final wording of the submission.

### Conflict of Interest Statement

The authors declare that the research was conducted in the absence of any commercial or financial relationships that could be construed as a potential conflict of interest.
